# Locally delivered GLP-1 analogues liraglutide and exenatide enhance microvascular perfusion in individuals with and without type 2 diabetes

**DOI:** 10.1007/s00125-019-4918-x

**Published:** 2019-06-16

**Authors:** Myo Myo Aung, Kate Slade, Leighton A. R. Freeman, Katarina Kos, Jacqueline L. Whatmore, Angela C. Shore, Kim M. Gooding

**Affiliations:** 1 Diabetes and Vascular Medicine, Institute of Biomedical and Clinical Sciences, University of Exeter Medical School, Barrack Road, Exeter, EX2 5AX UK; 2 Endothelial Cell Biology Group, University of Exeter Medical School, Exeter, UK; 3 Obesity Research Group, University of Exeter Medical School, Exeter, UK; 4 National Institute of Health Research Exeter Clinical Research Facility, Royal Devon and Exeter NHS Foundation Trust, Exeter, UK

**Keywords:** Exenatide, Glucagon-like peptide-1 analogues, Liraglutide, Microvascular perfusion, Obesity, Type 2 diabetes

## Abstract

**Aims/hypothesis:**

Glucagon-like peptide-1 (GLP-1) analogues reduce the risk of macrovascular disease in diabetes; however, little is known about their microvascular effects. This research examined the microvascular actions of the GLP-1 analogues liraglutide and exenatide in individuals with and without type 2 diabetes (study 1). It also explored the involvement of the GLP-1 receptor (study 2) and the nitric oxide pathway in mediating the microvascular effects of the analogues.

**Methods:**

Trial design: Studies 1 and 2 had a randomised, controlled, double-blind study design. Study 1 participants, intervention and methods: three participant groups were recruited: individuals with well-controlled type 2 diabetes, and obese and lean individuals without diabetes (21 participants per group). Liraglutide (0.06 mg), exenatide (0.5 μg) and saline (154 mmol/l NaCl; 0.9%) control were microinjected into separate sites in the dermis (forearm) in a randomised order, blinded to operator and participant. Skin microvascular perfusion was assessed by laser Doppler perfusion imaging. Outcomes were stabilised response (mean skin perfusion between 7.5 and 10 min post microinjection) and total response (AUC, normalised for baseline perfusion). Perfusion response to GLP-1 analogues was compared with saline within each group as well as between groups. Study 2 participants, intervention and methods: in healthy individuals (*N* = 16), liraglutide (0.06 mg) and saline microinjected sites were pretreated with saline or the GLP-1 receptor blocker, exendin-(9,39), in a randomised order, blinded to participant and operator. Outcomes were as above (stabilised response and total perfusion response). Perfusion response to liraglutide was compared between the saline and the exendin-(9,39) pretreated sites. In vitro study: the effects of liraglutide and exenatide on nitrate levels and endothelial nitric oxide synthase phosphorylation (activation) were examined using human microvascular endothelial cells.

**Results:**

Study 1 results: both analogues increased skin perfusion (stabilised response and total response) in all groups (*n* = 21 per group, *p* < 0.001), with the microvascular responses similar across groups (*p* ≥ 0.389). Study 2 results: liraglutide response (stabilised response and total response) was not influenced by pretreatment with exendin-(9,39) (70 nmol/l) (*N* = 15, one dataset excluded) (*p* ≥ 0.609). Liraglutide and exenatide increased nitrate production and endothelial nitric oxide synthase (eNOS) phosphorylation (*p* ≤ 0.020).

**Conclusions/interpretation:**

Liraglutide and exenatide increased skin microvascular perfusion in individuals with and without well-controlled diabetes, potentially mediated, at least in part, by NO.

**Trial registration:**

ClinicalTrials.gov NCT01677104.

**Funding:**

This work was supported by Diabetes UK (grant numbers: 09/0003955 and 12/0004600 [RW and JM Collins Legacy, Funded Studentship]).

## Introduction

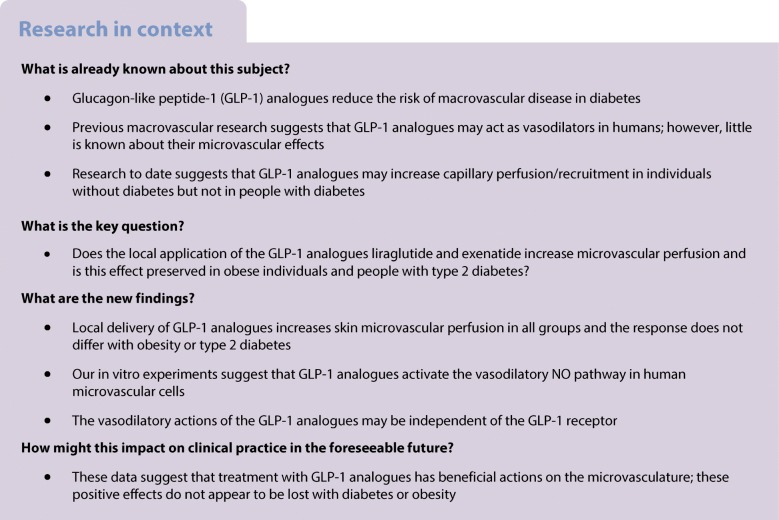



Diabetes is associated with vascular complications, which reduce the quality of life of individuals. Therapies that reduce the progression of these vascular complications are critically needed. Glucagon-like peptide-1 (7,36) amide (hereafter referred to as GLP-1) analogues are licensed therapies for type 2 diabetes, aiding glycaemic control by several mechanisms including stimulating postprandial release of insulin and reducing glucagon secretion [1]. Since there is growing evidence that they have beneficial cardiovascular properties, it is important to fully elucidate the effect of GLP-1 analogues on the cardiovascular system in diabetes. This would aid our understanding of their potential clinical role in protecting the vasculature, particularly since they are not first or second line treatments and may guide clinicians in treatment decisions.

The Liraglutide Effect and Action in Diabetes: Evaluation of Cardiovascular Outcome Results (LEADER) and the Trial to Evaluate Cardiovascular and Other Long-term Outcomes with Semaglutide in Subjects with Type 2 Diabetes (SUSTAIN-6) outcome trials demonstrated that GLP-1 analogues, compared with placebo, increased time to cardiovascular event in individuals with type 2 diabetes [2, 3]. The underlying mechanisms are not fully known, although a potential contributing mechanism is a direct action of GLP-1 analogues on the macrovasculature [4].

The impact of GLP-1 analogues on the microvasculature is even less understood, partly due to the scarcity and lack of concordance of available research. An exploratory objective of the LEADER cardiovascular trial was to examine the impact of liraglutide on incidence of microvascular events; results suggest that liraglutide may have differential microvascular effects, reducing the incidence of nephropathy events but with no significant effect on retinopathy [2]; similar observations were seen in SUSTAIN-6 [3].

There are few studies examining the microvascular actions of GLP-1 and its analogues. It has been shown that acute, systemic administration of GLP-1 (physiological and supraphysiological levels) increases muscle and cardiac microvessel recruitment in young, healthy, lean individuals [5, 6]. Additionally, acute, systemic infusion of the GLP-1 analogue exenatide (therapeutic levels) increases the number of perfused dermal capillaries at rest and during reactive hyperaemia in young, healthy, overweight men [7]. Conversely, neither acute administration of exenatide nor short-term liraglutide (12 weeks) influenced capillary perfusion in individuals with type 2 diabetes (fasting and postprandial states) [8]. Interestingly, changes in vasomotion in the skin (dorsal surface of middle finger) were observed in these acute exenatide studies by Smits and colleagues; however, it is difficult to differentiate the systemic effects of exenatide (e.g. increase in heart rate and BP, and reduction in plasma glucose levels) from the local skin microvascular effects. For example, exenatide-induced perfusion changes in the vasomotion neurogenic domain in the fasting state were correlated with glucose, diastolic BP and heart rate in individuals with diabetes [8].

Systemic administration is a widespread impediment to interpreting GLP-1 analogue-related vascular studies, making it difficult to differentiate any direct vascular actions of the analogues from effects due to systemic endocrine (e.g. increased insulin levels), haemodynamic and glycaemic changes. For example, GLP-1 analogues increase circulating levels of insulin, which is known to influence microvascular function [9]. For instance, Tesauro et al [10] observed that acute administration of GLP-1 enhanced endothelial (in)dependent forearm blood flow responses in hyperinsulinaemic conditions in individuals with the metabolic syndrome but had no effect in the absence of hyperinsulinaemia. These difficulties can be overcome by the local, direct administration of the analogues. The skin microvasculature, associated with the coronary circulation [11] and risk of CHD [12], presents an accessible, relevant microvascular bed for examination.

Our limited understanding of the mechanisms underlying the vascular responses to GLP-1 analogues is complicated by the diverse models used and complexity of the potential incretin-influenced vascular pathways. Previous human-based research (macrovascular cells and digital reactive hyperaemia) proposed that GLP-1 analogue actions are GLP-1 receptor (GLP-1R) dependent [13, 14], as well as NO dependent [13–18] and/or independent [19]. Other proposed mediators in humans include K_ATP_ channels [20] and endothelin-1 pathways [15].

The only available study investigating the microvasculature suggested that an exenatide-induced increase in the number of perfused capillaries in healthy, overweight men (*n* = 10) was NO independent [7]. Thus, further research is needed to fully elucidate the effect of GLP-1 analogues on the microvasculature in diabetes to aid our understanding of their potential clinical role in protecting the microcirculation.

Primary aims of this study were to (1) examine the direct, local effect of the GLP-1 analogues, exenatide and liraglutide, on microvascular function; and (2) assess whether the microvascular effects of exenatide and liraglutide differ in lean and obese individuals and in individuals with type 2 diabetes. A secondary aim was to examine whether the GLP-1R and the NO pathways mediate the microvascular actions of GLP-1 analogues. A further aim was to explore whether the microvascular response to liraglutide is associated with clinical characteristics, which may help to identify which individuals show the greatest microvascular benefit.

## Methods

All studies followed the principles of the Declaration of Helsinki. Written, informed consent was obtained from all participants. Microvascular assessments were performed the morning following an overnight fast in a temperature-controlled laboratory with the participant in a relaxed, supine position within the National Institute of Health Research (NIHR) Exeter Clinical Research Facility. Participants were recruited via the Peninsula Research Bank, part of the NIHR Exeter Clinical Research Facility.

### Study 1: Examining the microvascular actions of exenatide and liraglutide in health and whether this response is altered by obesity and diabetes

#### Participants

Three participant groups were recruited (*n* = 21 per group): lean individuals (BMI ≤25.0 kg/m^2^); obese individuals (BMI ≥30.0 kg/m^2^); individuals with type 2 diabetes. For the lean and obese groups, exclusion criteria included: diabetes; cardiovascular disease (CVD); Raynaud’s disease; current treatment with any antihypertensive or lipid-lowering therapies.

For the type 2 diabetes group, individuals on stable diabetes medication (minimum of 3 months) or diet control only were recruited. Exclusion criteria included: insulin or sulfonylurea treatment; known CVD; proliferative retinopathy; advanced nephropathy (macroalbuminuria); uncontrolled diabetes (HbA_1c_ >8.5%/69 mmol/mol); previous GLP-1 analogue or dipeptidyl peptidase-4 inhibitor treatment. The study was approved by the National Research Ethics Committee South West – Exeter (NREC-SWE) (11/SW/0195) and was registered on ClinicalTrials.gov (NCT01677104).

#### Methods

The study had a randomised, controlled, double-blind study design.

#### Participant characterisation and biochemical assessment methods

Participant characterisation: Body composition assessments included height, weight and waist-to-hip ratio. Waist circumference was measured (end of expiration) midway between the costal margin and iliac crest. Hip circumference was measured at the widest horizontal circumference. All measurements were repeated three times and the mean value taken. BP was taken using a semi-automatic device (Dinamap, Critikon, FL, USA) five times at 1 min intervals; the mean of the last three measurements represented BP. A timed, overnight urine sample was collected by all participants to assess for microalbuminuria (AER >20 μg/min). Neuropathy was assessed using monofilaments (Semmes Weinstein 10 g monofilament, Owen Mumford, Woodstock, UK) over six sites on each foot. A score of ≤3 out of 6 in either foot was classed as significant neuropathy.

Biochemical assessments: Plasma glucose, creatinine, triacylglycerols, total cholesterol and HDL levels, and urinary albumin were determined using Modular Analytics, Roche P800 (Roche Diagnostics, Mannheim, Germany). LDL was calculated using the Friedewald formula. HbA_1c_ was determined with the gold standard ion-exchange method (Tosoh G8 HPLC Analyzer, Tosoh Bioscience, San Francisco, CA, USA). Insulin samples were analysed using the Roche E170 chemiluminescent immunoassay (Roche Diagnostics). HOMA insulin resistance was calculated using fasting blood glucose and plasma insulin (HOMA calculator V 2.2.3, 2004, University of Oxford, Diabetes Trial Unit, Oxford, UK). eGFR was calculated using the Modification of Diet in Renal Disease (MDRD) equation [21].

#### Microvascular assessment

Participants with diabetes abstained from medication on the study morning. Fasting blood samples were taken upon arrival.

#### Microinjection protocol

The GLP-1 analogues (one-tenth of the lowest treatment dose, exenatide [0.5 μg; Lilly, Basingstoke, UK] and liraglutide [0.06 mg; Novo Nordisk, Gatwick, UK]), acetylcholine (ACh, 1% [10 mg/ml]; Miochol-E, Bausch & Lomb, Kingston-Upon-Thames, UK) (positive endothelial function control) and 0.9% saline (154 mmol/l NaCl; Fannin, Dublin, UK) (injection trauma control) were delivered by microinjection on the same visit. Exenatide was diluted (1:5) with saline to obtain 0.5 μg. The order of test sites was blinded and randomised using a random number generator (http://www.stattrek.com).

A black adhesive collar was attached to the volar aspect of the forearm to delineate the region of interest (ROI) (1.54 cm^2^) for each microinjection site. Each site was at least 2 cm apart, avoiding visible veins, skin lesions, freckles and hair. A sterile, disposable insulin syringe (30G, BD Microfine, Becton Dickinson, Dublin, Ireland), bent 90^o^, was used to deliver 10 μl of the test substance into the dermis of the ROI. The skin perfusion response was assessed by laser Doppler perfusion imaging (LDPI) (PIM 3.0, Perimed, Järfälla, Sweden) at baseline (resting perfusion) and then every 30 s for 10 min post injection. The skin perfusion response is reported as the stabilised response (mean perfusion 7.5–10 min post injection) and total response (AUC, normalised for resting perfusion). The stabilised response examines the response to the substance of interest following the resolution of the injection trauma perfusion response. Intraparticipant CV for the stabilised response was 9.7% (mean ± SD: 1.40 ± 0.13 *V*) for liraglutide and 8.7% (1.00 ± 0.09 *V*) for exenatide, calculated from one lean individual on three separate occasions. This protocol (doses and assessment time) was informed by preliminary experiments. Blood glucose was regularly monitored throughout the study.

### Study 2: Examining the role of the GLP-1R in mediating the microvascular actions of liraglutide

#### Participants

Sixteen healthy (no diabetes, hypertension or CVD), lean individuals (BMI ≤25 kg/m^2^), >18 years, were recruited. The exclusion criteria were the same as for the lean group in study 1. No participants took part in both study 1 and study 2. The study was approved by the NREC-SWE (14/SW/0093).

#### Study design

The study had a randomised, controlled, double-blind study design. Participant characterisation, biochemical assessments and study conditions were as for study 1.

#### Microinjection protocol

To examine the impact of GLP-1R inhibition on microvascular response to liraglutide, the ROI was pretreated with exendin-(9,39) (70 nmol/l) (Bachem, Bubendorf, Switzerland) using a double microinjection protocol. Exendin-(9,39) (10 μl) was initially microinjected (first microinjection), followed 60 s later by liraglutide (10 μl, 0.06 mg) (second microinjection) ~1 mm from the first microinjection site (referred to as Exendin-(9.39) liraglutide site). Three further sites were treated with (1) saline (0.9%) followed by liraglutide (referred to as liraglutide site); (2) exendin-(9,39) followed by saline (referred to as exendin-(9,39) site); and (3) saline followed by saline (microinjection trauma control, referred to as saline site). The order of test sites was blinded and randomised as above.

Microinjection, data acquisition and analysis were performed as described for study 1. The skin perfusion response of each site was assessed by LDPI at baseline, immediately after the first microinjection and every 30 s for 10 min following the second microinjection.

### In vitro study: Examining the role of NO in mediating the microvascular actions of GLP-1 analogues

The role of NO in mediating the microvascular actions of GLP-1 analogues was examined in cultured human microvascular endothelial cells (HCMEC/D3 cell line) [22] by assessing the effects of exenatide (100 pmol/l) (Isca Biochemicals, Exeter, UK) and liraglutide (10 nmol/l) (Isca Biochemicals) on endothelial nitric oxide synthase (eNOS) activation (phosphorylation) and nitrate levels.

#### eNOS phosphorylation

Cells were treated with medium only (control), exenatide or liraglutide for 10 min (*n* = 8 per condition) and eNOS phosphorylation was determined using a commercially available ELISA kit (eNOS Phospho-Ser 1176 [catalogue No.: OKAG01931], Aviva Systems Biology, San Diego, CA, USA). Phosphorylated levels were normalised to total eNOS and data are expressed as percentage of control.

#### Nitrate levels

Cells were treated with medium only (control), exenatide or liraglutide for 24 h (*n* = 9 per condition). Supernatants were then collected and stored for nitrate analysis using a Sievers Nitric Oxide Analyzer (Sievers NOA 280, Analytix, Tyne & Wear, UK) [23]. Results are expressed as percentage of control (medium only).

### Statistical analysis

Data are presented as mean ± SD or as median (25th–75th percentile) if data were not normally distributed. Significance was defined as *p* ≤ 0.05. In keeping with Cupples et al [24] and Rothman [25], significance is reported without adjustment for multiple testing.

Study 1: 21 individuals per group were recruited, enabling the study to detect a 1 SD difference between groups and a 0.70 SD within-participant difference at 90% power. For within-group analysis, ANOVAs for repeated measures or Friedman’s test, depending on normality, were initially used. Post hoc testing used either paired *t* tests or Wilcoxon signed rank tests to determine where the difference(s) were (saline vs exenatide, liraglutide or ACh). For between-groups analysis, one-way ANOVAs or Kruskal–Wallis test were initially performed. Post hoc testing used either a Student’s *t* test or a Mann–Whitney *U* test.

Study 2: 16 individuals were recruited, enabling the study to detect a 0.8 SD within-participant difference at 90% power. Paired *t* test or Wilcoxon signed rank test, depending on normality of data, was used to determine whether the GLP-1R inhibition altered the microvascular response to liraglutide (liraglutide site vs exendin-(9,39) liraglutide site).

To examine whether the microvascular actions of liraglutide are associated with clinical and metabolic characteristics (age, body composition, BP, glycaemic control and lipid profile), data from both study 1 and study 2 were merged. The stabilised response to liraglutide across the merged cohorts was initially examined using Spearman’s correlation test. Significant associations from univariate analysis, using the variable with the strongest *r* for each class of characteristics (e.g. BP or lipid profile variable), were further explored using linear regression, adjusting for potential confounding factors (sex and stabilised response to saline control site).

In vitro study: the Mann–Whitney *U* test was used to compare the responses to exenatide and liraglutide with the responses to control in the in vitro experiments.

## Results

### Study 1

Sixty-three participants completed the study. In the type 2 diabetes group, diabetes was controlled by diet alone in five (24%) and by metformin in 16 (76%) participants. Median duration of diabetes was 7 (25th–75th percentile: 3–9) years, and 86% of participants with diabetes were taking cholesterol-lowering tablets and 57% antihypertensive treatment. None of the participants (all groups) showed any evidence of microalbuminuria, advanced retinopathy or significant neuropathy.

HbA_1c_ and fasting glucose levels were within the normal range for all participants in the obese group. Insulin and HOMA levels were significantly higher in participants with obesity and diabetes than in lean participants (Table 1). BMI in the obese group was also significantly higher than in the diabetes group.

**Table 1:** Clinical characteristics of the lean, obese and type 2 diabetes groups in study 1

Characteristic	Lean group	Obese group	Type 2 diabetes group	*p* value^a^
Sample size (% male)	21 (57)	21 (29)	21 (43)	
Age (years)	65 (54–70)	67 (52–70)	70 (64–71)	0.064
BMI (kg/m^2^)	23.00 (22.00–24.00)	33.00 (31.50–38.00)***	30.00 (26.25–33.00)***^,†††^	˂0.001
Waist circumference (cm)	81.69 ± 6.64	104.93 ± 10.55***	99.73 ± 9.99***	˂0.001
Waist/hip ratio	0.83 (0.80–0.88)	0.86 (0.83–0.97)*	0.91 (0.86–0.99)***	˂0.001
Systolic BP (mmHg)	127 (118–138)	140 (134–154)*	141 (134–155)*	0.037
Diastolic BP (mmHg)	76 (70–87)	81 (75–88)	77 (71–86)	0.221
Mean arterial pressure (mmHg)	92 ± 10	99 ± 10	99 ± 12	0.082
HbA_1c_ (mmol/mol)	39.00 ± 2.02	38.8 ± 3.6	48.9 ± 6.9***^,†††^	˂0.001
HbA_1c_ (%)	5.7 ± 2.3	5.7 ± 2.5	6.6 ± 2.8***^,†††^	
Fasting blood glucose (mmol/l)	4.80 (4.60–5.33)	5.30 (5.05–5.50)*	7.03 (6.01–7.74)***^,†††^	˂0.001
Fasting plasma insulin (pmol/l)	29.33 (22.59–37.19)	64.20 (54.64–176.80)***	84.50 (49.30–122.00)***	<0.001
HOMA (insulin resistance)	0.60 (0.40–0.75)	1.20 (1.00–2.05)***	1.60 (1.00–2.30)***	<0.001
Fasting total cholesterol (mmol/l)	5.68 ± 1.01	5.38 ± 0.90	4.15 ± 0.96***^,†††^	˂0.001
HDL (mmol/l)	1.67 (1.50–2.11)	1.38 (1.10–1.70)*	1.41 (1.26–1.74)*	0.016
LDL (mmol/l)	3.22 ± 0.86	3.02 ± 0.99	2.07 ± 0.89**^,††^	0.002
Fasting triacylglycerols (mmol/l)	0.83 (0.66–1.09)	1.30 (1.03–1.76)**	1.14 (0.87–1.69)*	0.004
eGFR (ml/min/1.73 m^2^)	84.0 (71.0–86.0)	71.0 (62.0–82.0)	81.5 (68.3–89.0)	0.552

Data presented as mean (SD) or median (25th–75th percentile)

^a^*p* value for between-group analysis across all three groups

*p<0.05, **p<0.01, ***p<0.001 vs lean group

^††^*p*<0.01, ^†††^*p*<0.001 vs obese group

#### The direct, local effect of GLP-1 analogues, exenatide and liraglutide, on skin perfusion

**Fig. 1:**
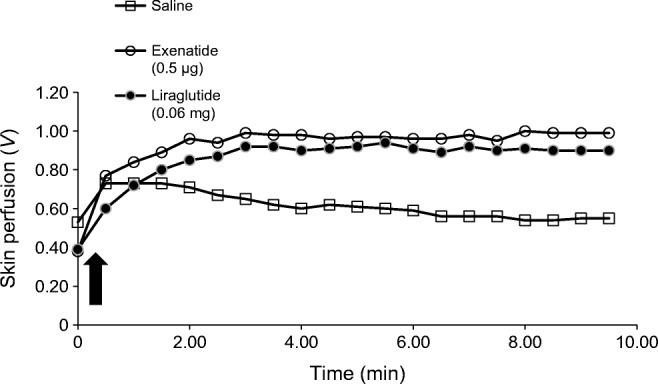
Representative skin perfusion response to microinjection of the GLP-1 analogues exenatide and liraglutide, compared with saline control, in a lean individual; the graph represents a typical pattern of response that was observed across all participants (*n*=63). The arrow denotes time of microinjection

All microinjections, including saline, caused an initial increase in skin perfusion due to the injection trauma. The response to saline decays over time, presumably due to resolution of this trauma response (representative graph in Fig. 1). Exenatide, liraglutide and ACh significantly increased skin perfusion compared with control in all participant groups (*p* values <0.001). The microinjection protocol was well tolerated by all participants. Blood glucose remained in the normal range throughout the microinjection protocol.

#### The effects of obesity and diabetes on the microvascular effects of exenatide and liraglutide

**Fig. 2:**
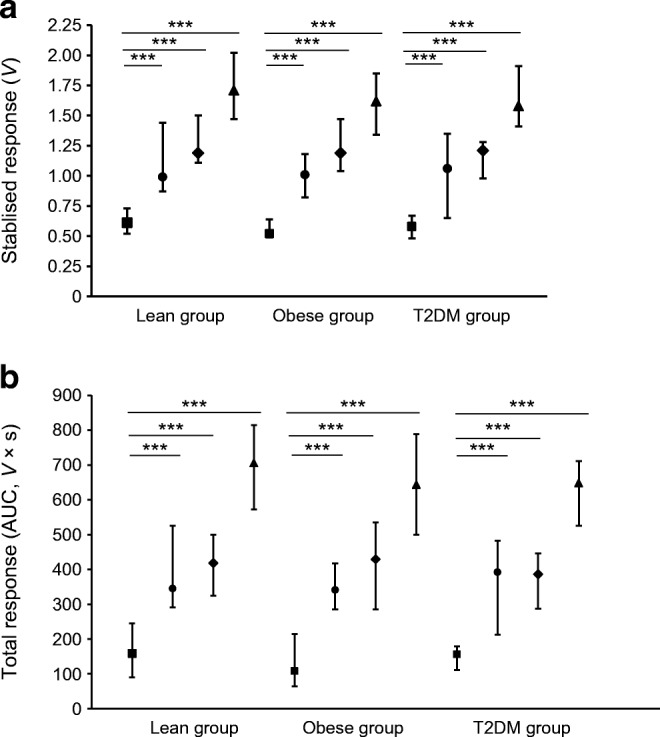
Skin perfusion response to microinjection of exenatide, liraglutide, ACh and saline in the lean, obese and type 2 diabetes groups (*n*=21 in each group). (**a**) Stabilised response and (**b**) total perfusion response to saline (squares), exenatide (circles), liraglutide (diamonds) and ACh (triangles). Data are presented as median (25th–75th percentile). The saline response was significantly lower than the responses to exenatide, liraglutide and ACh, respectively (stabilised response [**a**] and total response [**b**]) in all participant groups (***p<0.001, Wilcoxon signed rank tests). There was no difference in the response to exenatide, liraglutide or ACh between the participant groups. T2DM, type 2 diabetes

The microvascular responses to exenatide, liraglutide and ACh (stabilised response and total response) were comparable between the three groups of participants (stabilised and total response to exenatide and liraglutide: *p* values ≥ 0.456 and ≥ 0.389, respectively; stabilised and total response to ACh: *p* = 0.332 and 0.250, respectively) (Fig. 2).

### Study 2

**Fig. 3:**
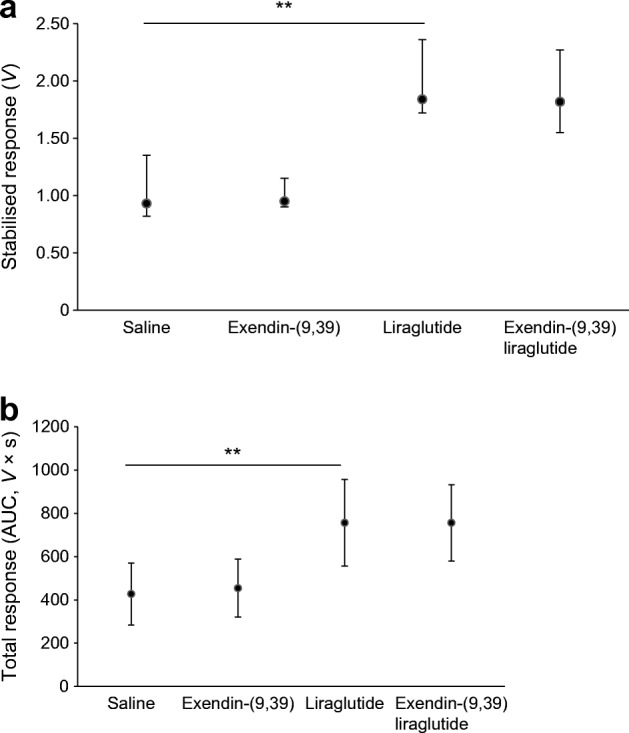
GLP-1R inhibition does not alter the skin microvascular response to liraglutide in healthy individuals. Each participant (*n*=15) had 4 treatment sites, each receiving two microinjections: saline site (saline followed by saline); exendin-(9,39) (exendin-(9,39) followed by saline); liraglutide (saline followed by liraglutide); exendin-(9,39) liraglutide (exendin-(9, 39) followed by liraglutide). (**a**) Stabilised response, data presented as median (25th–75th percentile). (**b**) Total response, data presented as mean (SD). The skin perfusion response at the saline site was significantly lower than the response (stabilised and total) at the liraglutide site (**p<0.01 by Wilcoxon signed rank test for stabilised response; by paired *t* test for total response). Pretreatment by microinjection of exendin-(9,39) did not alter the microvascular response to liraglutide (liraglutide site vs exendin-9,39 liraglutide site, *p*≥0.609 by Wilcoxon signed rank test for stabilised response; by paired *t* test for total response)

Fifteen participants completed the study (one excluded owing to technical issues) (Table 2). Liraglutide significantly increased skin perfusion (stabilised response and total response median [25th–75th percentile]: 1.84 [1.72–2.36] *V* and 708 [630–853] *V* × s, respectively) compared with the saline control site (0.93 [0.82–1.35] *V* and 395 [320–506] *V* × s) (*p* values = 0.001) and the response to liraglutide was not altered by pretreatment with exendin-(9,39) (exendin-(9,39) liraglutide site stabilised and total response: 1.82 [1.55–2.27] *V* and 761 [640–854] *V* × s, respectively, *p* ≥ 0.609) (Fig. 3).

**Table 2:** Characteristics of participants in study 2

Variable	Value
Sample size (% male)	16 (53)
Age (years)	32 (22–36)
BMI (kg/m^2^)	22.1 ± 1.2
Waist circumference (cm)	80.7 ± 7.0
Waist/hip ratio	0.82 ± 0.05
Systolic BP (mmHg)	115 ± 10
Diastolic BP (mmHg)	66 ± 6
Mean arterial pressure (mmHg)	82 ± 7
HbA_1c_ (mmol/mol)	33.6 ± 3.0
HbA_1c_ (%)	5.2 ± 0.3
Fasting blood glucose (mmol/l)	4.93 ± 0.31
Fasting total cholesterol (mmol/l)	4.05 ± 0.51
HDL (mmol/l)	1.60 ± 0.35
LDL (mmol/l)	2.05 ± 0.62
Fasting triacylglycerols (mmol/l)	0.85 (0.73–1.20)

Data are presented as mean ± SD or median (25th–75th percentile)

#### Relationship between the response to liraglutide and clinical and metabolic characteristics

As the response to GLP-1 analogues was not altered by diabetes or obesity in study 1, all of the participants from studies 1 and 2 were collated to examine whether the response to liraglutide was associated with clinical and metabolic characteristics. This resulted in a cohort of 78 participants (44% male) with an age range of 21–85 years (Table 3).

**Table 3:** Characteristics of participants merged from both study 1 and study 2

Variable	Value
Sample size (% male)	78 (44%)
Age (years)	64 (46, 70)
BMI (kg/m^2^)	24.9 (23.0–32.0)
Waist circumference (cm)	90.3 (81–103)
Waist/hip ratio	0.86 (0.81–0.92)
Systolic BP (mmHg)	135 (124–147)
Diastolic BP (mmHg)	77 ± 9
Mean arterial pressure (mmHg)	97 ± 11
HbA_1c_ (mmol/mol)	39 (37–44)
HbA1c (%)	5.7 (5.5–6.2)
Fasting blood glucose (mmol/l)	5.3 (4.8–6.0)
Fasting total cholesterol (mmol/l)	4.91 ± 1.13
HDL (mmol/l)	1.56 (1.33–1.91)
LDL (mmol/l)	2.66 ± 0.99
Fasting triacylglycerols (mmol/l)	1.06 (0.75–1.47)

Data are presented as mean ± SD or median (25th–75th percentile)

The stabilised response to liraglutide was negatively associated with age (Spearman’s correlation: *r*_s_ = −0.679, *p* < 0.001), BP (systolic BP: *r*_s_ = −0.422, *p* < 0.001; diastolic BP: *r*_s_ = −0.312, *p* = 0.005; mean arterial pressure: *r*_s_ = −0.382, *p* = 0.001), body composition (waist circumference: *r*_s_ = −0.302, *p* = 0.007; waist/hip ratio: *r*_s_ = −0.251, *p* = 0.026) and glycaemic control (fasting glucose: *r*_s_ = −0.298, *p* = 0.010; HbA_1c_: *r*_s_ = −0.494, *p* < 0.001). There was no association between response to liraglutide and the lipid profile.

The observed associations of liraglutide (stabilised response) with age, body composition (waist circumference), BP (systolic) and glycaemic control (HbA_1c_), adjusting for sex and response to saline control, were separately explored using linear regression (Table 4). Both age and HbA_1c_, after adjustment for saline response and sex, were individually associated with the response to liraglutide (Table 4). When age, HbA_1c_, sex and response to saline control were included in the model (combined model) they accounted for 50.3% of the variance of the response to liraglutide (adjusted *R*^2^ = 0.503, *F* = 20.496, *p* < 0.001). Age but not HbA_1c_ was independently associated with the stabilised response to liraglutide in this combined model (Table 4).

**Table 4:** Linear regression analysis of stabilised response to liraglutide and clinical and metabolic characteristics

Variable	Unstandardised β coefficient (SE)	Standardised β coefficient	*p* value
Univariate analysis adjusting for sex and response to saline control			
Age	−0.016 (0.003)	−0.568	<0.001
Waist	−0.004 (0.003)	−0.125	0.230
SBP	−0.003 (0.002)	−0.144	0.178
HbA_1c_	−0.019 (0.006)	−0.285	0.003
Combined model (age, HbA_1c_, sex and response to saline control)			
Age	−0.015 (0.003)	−0.542	<0.001
HbA_1c_	−0.003 (0.007)	−0.040	0.687

### In vitro study

**Fig. 4:**
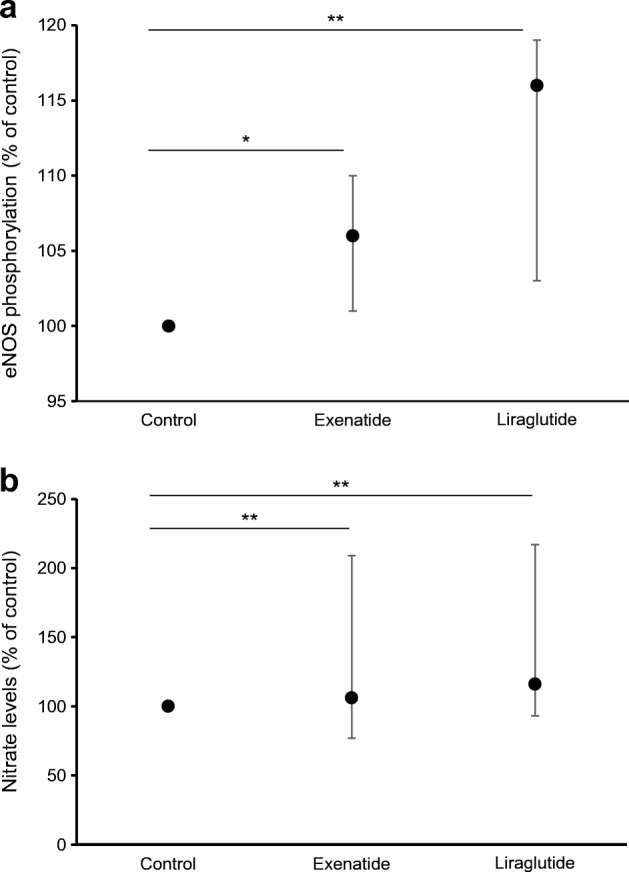
Exenatide and liraglutide increase eNOS phosphorylation and nitrate levels. (**a**) eNOS phosphorylation: after initial starvation, human microvascular endothelial cells (HCMEC/D3s) were treated with exenatide and liraglutide for 10 min. Controls were treated with 0.1% BSA medium only. Phosphorylation data were normalised to total eNOS (*n*=8). (**b**) Nitrate levels: HCMEC/D3s were treated with exenatide and liraglutide for 24 h (*n*=9). Controls were treated with medium only. For both (**a**) and (**b**), data are expressed as percentage of control, with control set at 100%, and are presented as median (25th–75th percentile). *p<0.05, **p<0.01 vs control, Wilcoxon sign rank test.

Liraglutide and exenatide significantly increased eNOS phosphorylation compared with control (liraglutide median [25th–75th percentile]: 116 [103–119]%, *p* = 0.001; exenatide: 106 [101–110]%, *p* = 0.020; control: 100%) and nitrate levels compared with control (liraglutide median [25th–75th percentile]: 201 [178–302]%, *p* = 0.002; exenatide: 169 [140–272]%, *p* = 0.002; control: 100%) (Fig. 4).

## Discussion

Here, we provide the first evidence of a direct vasodilatory action of GLP-1 analogues in the microcirculation in lean individuals in vivo. Importantly, we also demonstrate that this response is not attenuated in obesity and type 2 diabetes, conditions associated with microvascular impairments [26, 27]. Furthermore, this research suggests that this increase in microvascular perfusion may be mediated, at least partially, by NO but not via the GLP-1R.

These data suggest that the GLP-1 analogues have a direct vasodilatory effect on the microvasculature (arterioles, venules and capillaries). In contrast, the only previously reported research in this area examined the systemic effects of exenatide or liraglutide on microvascular perfusion in humans, in vivo [7, 8]; an increase in the number of perfused capillaries at rest and during post-occlusive reactive hyperaemia was observed in response to exenatide (continuous i.v. infusion resulting in therapeutic levels) in young (20–27 years), overweight, male participants [7]. Whether this increase in capillary perfusion resulted from a direct action of exenatide is unclear, but results from our study would suggest that this is the case.

We also showed that the microvascular actions of exenatide and liraglutide are maintained in obesity and type 2 diabetes. In contrast, Smits et al [8] observed no changes in capillary perfusion with either acute or short-term treatment with GLP-1 analogues in individuals with relatively well-controlled diabetes (HbA_1c_ mean ± SEM: 7.3 ± 0.3% [mean: 56.3 mmol/mol]; age: 62.8 ± 6.9 years). The participants with type 2 diabetes in the current study (study 1) were slightly older (mean [25th–75th percentile]: 70 [64–71] years) with marginally better glycaemic control (6.6 ± 2.8% [48.9 ± 6.9 mmol/mol]) than those recruited by Smits et al [8]. Since HbA_1c_ was not related to the microvascular responsiveness to GLP-1 analogues and participants were slightly older in our study, when age was negatively related to response, it is unlikely that these variations in participant characteristics could explain the discrepancies between the current study and that of Smits et al [8].

Potential reasons for the observed differences between the diabetes groups in our study and the study by Smits et al [8] may be the delivery route (local vs systemic) and dose (local, dermal delivery at one-tenth minimal treatment dose vs systemic, i.v. administration of therapeutic levels). As detailed earlier, systemic application of GLP-1 analogues at therapeutic doses may result in systemic endocrine, haemodynamic and glycaemic changes, potentially influencing microvascular function including vasomotion [7, 8]. In study 1, there was a small drop in blood glucose and insulins levels across the study visit (glucose: ~2%; insulin: 18–33%), reflecting the participants’ fasting state. Importantly, glucose remained in the normal glycaemic range for all participants. Additionally, the site and imaging techniques differed between the two studies (i.e. direct visualisation of digital dermal capillaries vs LDPI of forearm skin microcirculation).

The strength of the current study is that GLP-1 analogues were delivered locally, ensuring direct microvascular actions without the complication of systemic effects. However, it is important to note that the participants with diabetes had well-controlled glycaemia, no evidence of clinical microvascular complications and good endothelial function, as assessed by ACh microinjection. Whether this conserved response to exenatide and liraglutide in obesity and diabetes reflects that this pathway is intact in these populations, or whether this is partially explained by the relative healthiness of our participants, is unclear. However, the results of the LEADER cardiovascular study [2], which observed vascular benefits in individuals with type 2 diabetes and established, or risk factors for, CVD, may imply that this vascular pathway is intact, although further research is needed to confirm this.

Age was negatively associated with the response to liraglutide. Microvascular function declines with age [28]; however, whether the observed association is due to age-related impairment of microvascular function or specifically due to the GLP-1 pathway is unclear. Since liraglutide is an effective glycaemic treatment in the elderly (>65 years) [29], the observed relationship may relate more to a natural decline in microvascular function with age. This is supported by the association between age and response to ACh (unstandardised β [SE]: −0.011 [0.005]; standardised β: −0.294, *p* = 0.003) in this study. Collectively, these data highlight the need to take age into account in human, in vivo studies.

GLP-1Rs have been described in human microvascular endothelial cells [30, 31]. However, the current study suggests that the local microvascular actions of the GLP-1 analogues are GLP-1R independent in healthy humans. This is in contrast to observations by Koska et al [13], who observed an impaired exenatide-induced increase in digital reactive hyperaemia with systemic blockade of the GLP-1R with exendin-(9,39) in participants with impaired glucose tolerance or diet-controlled diabetes. However, the 70 nmol/l concentration used in study 2 was similar to plasma levels of exendin-(9,39) previously shown to abolish GLP-1-induced insulin secretion and reduce plasma glucose levels in the hyperglycaemic state in healthy participants (plasma mean [SE]: 53 [4] nmol/l) [32].

The NO pathway was upregulated (activation/phosphorylation of eNOS and increased production of nitrate) in human microvascular endothelial cells by both exenatide and liraglutide, suggesting NO involvement in their microvascular actions. This is in agreement with previous studies on the human macrovasculature [14, 16], e.g. Koska et al observed a comparable increase in eNOS phosphorylation in human coronary artery endothelial cells [13], but this is the first demonstration in human microvascular endothelial cells. Interestingly, Smits et al observed that exenatide-induced increases in capillary perfusion in healthy, overweight individuals [7] were not modulated by NOS blockade. Since both studies used GLP-1 analogues within their therapeutic ranges, this may reflect differences between in vivo and in vitro studies and/or the heterogeneity of endothelial cells [33]. It is unlikely that the acute nature of the intervention influenced the observations by Smits et al [8] as the current study demonstrated that acute treatment (10 min) with GLP-1 analogues increased eNOS activation. The NO pathway may be impaired with obesity [26], raising the possibility that this pathway was downregulated and thus unresponsive in the overweight group recruited by Smits et al [7]. However, the observation that microvascular endothelial function was not impaired in the obese group in the current study suggests that this is not the case. Further research is needed to clarify the involvement of the GLP-1R and the NO pathway in mediating the microvascular actions of GLP-1 analogues, particularly in different populations in vivo.

Study limitations: This study may not be applicable to the general diabetes population as only individuals with uncomplicated, well-controlled diabetes were recruited. Additionally, study 2 could have benefited from the inclusion of a control to demonstrate successful GLP-1R inhibition and/or systemic delivery of exendin-(9,39). This research would also be strengthened by extending the NO investigations to examine the effect of NOS blockade in humans, in vivo.

Collectively, our data show that local administration of liraglutide and exenatide increases skin perfusion in lean and obese individuals as well as those with well-controlled, complication-free, type 2 diabetes. It seems likely that this increase in skin perfusion is mediated, at least partially, by an NO-dependent mechanism and independently of the GLP-1R. However, further research is needed to determine whether such local microvascular benefit is preserved in individuals with type 2 diabetes and established microvascular complications or with a systemic increase in GLP-1 levels, such as with clinical treatment with GLP-1 analogues.

## Data Availability

The datasets generated during and/or analysed during the current study are available from the corresponding author on reasonable request.

## Abbreviations

ACh: Acetylcholine
CVD: Cardiovascular disease
eNOS: Endothelial nitric oxide synthase
GLP-1: Glucagon-like peptide-1
GLP-1R: Glucagon-like peptide-1 receptor
LDPI: Laser Doppler perfusion imaging
LEADER: Liraglutide Effect and Action in Diabetes: Evaluation of Cardiovascular Outcome Results
NIHR: National Institute of Health Research
NREC-SWE: National Research Ethics Committee South West – Exeter
ROI: Region of interest
SUSTAIN-6: Trial to Evaluate Cardiovascular and Other Long-term Outcomes with Semaglutide in Subjects with Type 2 Diabetes
